# MicroRNA Profiles in Spontaneous Decidualized Menstrual Endometrium and Early Pregnancy Decidua with Successfully Implanted Embryos

**DOI:** 10.1371/journal.pone.0143116

**Published:** 2016-01-06

**Authors:** Yu Wang, Yang Lv, Shujun Gao, Yuanyuan Zhang, Jiajia Sun, Chunling Gong, Xiujuan Chen, Guangpeng Li

**Affiliations:** 1 The Key Laboratory of National Education Ministry for Mammalian Reproductive Biology and Biotechnology, Inner Mongolia University, Hohhot, China; 2 Department of Gynecology and Obstetrics, Inner Mongolia Medical University affiliated hospital, Hohhot, China; 3 College of Life Sciences, Inner Mongolia University, Hohhot, China; South China Agricultural University, CHINA

## Abstract

To comparatively analyze the human microRNA (miRNA) profiles between spontaneous decidualized menstrual endometrium and early pregnancy decidua by an in-depth sequencing of miRNAs. The specific miRNAs expressed at conception might be involved in pregnancy establishment and expression of let-7f-5p and let-7g-5p was experimentally up-regulated or inhibited to assess the effect on the expression of IGF2BP-1 and IGF2R in vitro, respectively. Samples of endometria and deciduas were obtained from 25 women who suffered from tubal or male factor subfertility and from 35 early pregnant women who underwent pregnancy termination at 6–8 weeks gestation were irrespectively collected and comparatively analyzed by miRNA sequencing and differential expression of known and novel miRNAs was analyzed using bioinformatics. The 2042 miRNA expression was analyzed in the study and the differential expression of six miRNAs was validated by qRT-PCR. The expression of four miRNAs in decidua samples was down-regulated (miR-34c, miR-92a, miR-181a-5p, and miR-191), whereas the expression of miR-10a-5p and let-7f-5p was significantly up-regulated. The expression of IGF2BP-1 and IGF2R declined and increased with overexpression and inhibition of let-7f-5p and let-7g-5p, respectively. Changes in the expression of particular miRNAs might play a role in the physiology of decidualization following successful embryo implantation, ultimately resulting in continuous decidualization.

## Introduction

MicroRNAs (miRNAs) are a class of small noncoding RNAs that regulate gene expression and play fundamental roles in many biological processes, including cell differentiation and proliferation. In general, miRNAs inhibit translation or induce mRNA degradation by binding to the 3’ untranslated region of the target mRNA. To date, over 2,000 human miRNAs have been registered in miRBase v19.0 [1, 2].

Abnormal miRNA expression is associated with a number of benign gynecologic conditions, malignancies, and fertility disorders [3]. The abundance of specific miRNAs as well as relative differences in expression level can be assessed through miRNA sequencing. Several independent studies have profiled miRNA expression in secretory and proliferative endometria [4–6]. Other studies have shown that differences in miRNA expression occur during the implantation window in the secretory endometrium of patients either with or without endometriosis [7, 8]. However, to date, no reports have described the miRNA profiles of the spontaneous decidualized menstrual endometrium and early pregnancy decidua.

Menstruation is triggered by a decline in the level of circulating progesterone and involves the periodic discharge of blood and mucosal tissue, which consists of the decidualizing superficial endometrium. In response to declining progesterone levels, spontaneous decidualization leads to menstrual shedding and subsequent regeneration of the endometrium. In contrast to the case with most mammals, embryo implantation is not required for decidualization of the human endometrium. Furthermore, menstruation and cyclic regeneration of the endometrium involve recruitment of stem cells, imparting an intrinsic capacity for adaptation of the decidual response to maximize reproductive success. Once initiated, decidualization proceeds through distinct phenotypic phases involving endometrial receptivity, embryo selection, and ultimately, resolution of pregnancy at pregnancy initiation [9, 10].

Based on our previous study [11], we hypothesized that various miRNAs might play a role in embryo implantation and decidualization. However, the profile of miRNA expression in the menstrual endometrium remains to be elucidated [12, 13]. Characterization of the miRNomes of the human early pregnancy decidua and menstrual endometrium via deep sequencing might thus identify those decidualization-associated miRNAs that are involved in embryo implantation [14, 15]. The aim of the present study, therefore, was to identify miRNAs that play an important role in embryo implantation by characterizing the expression of miRNAs in the human menstrual endometrium and early pregnancy decidua. The miRNA profile of the early pregnancy decidua was compared with that of the menstrual phase in order to obtain new insights into the roles of miRNAs at conception.

## Materials and Methods

### Tissues collection

Endometrial biopsies were performed on the second day of the menstrual cycle in 25 women who suffered from secondary infertility due to either tubal or male factors and who received treatment between October 2011 and April 2012 in the Reproductive Center of Inner Mongolia University Affiliated Hospital (Inner Mongolia, China). Participants ranged in age from 22 to 39 years (mean, 31 years). In addition, decidua tissue was collected from 35 women (mean age, 28.6 years; range, 19–39 years) with a normal pregnancy (based on ultrasound evidence of fetal heart activity) who underwent pregnancy termination at 6–8 weeks of gestation. Samples were collected for miRNA and associated target gene analysis. The decidua was separated from the trophoblast, and each tissue sample was immediately frozen in liquid nitrogen. Six normal-pregnancy deciduas and six menstrual endometria were collected for sequencing analysis from as previous mentioned. The women in each group from whom specimens were collected for sequencing did not differ significantly in age. The study was approved by the Institute Research Ethics Committee of the Inner Mongolia Medical University Affiliated Hospital, and written informed consent was obtained from all participants.

### Small RNA library construction and sequencing

Total RNA (including miRNAs) was extracted using RNAiso plus (Takara, Shiga, Japan). All samples passed the RNA quality control criteria for Solexa sequencing (RNA 28S:18S ≥1.5, RNA integrity number ≥8). The sequencing procedure was as described previously [11]. Small RNAs perfectly matching the miRBase precursor sequence and those identified as known miRNA editing polymorphisms in the miRBase 19.0 database [16, 17] were annotated and calculated. Mireap software [18] was used to predict novel miRNAs, as described previously [11].

### MiRNA target prediction and functional analysis

The function of an miRNA is ultimately determined by the genes that the miRNA targets and by the effect of the miRNA on the expression of those genes. The RNAhybrid software [19] was used to predict the target genes of the novel miRNAs identified in this study [20, 21].

### In vitro overexpression and inhibition of let-7f-5p and let-7g-5p

Human embryonic kidney (HEK293T) cells and human endometrial stromal cells (hESCs) were obtained from the American Type Culture Collection and cultured in DMEM (Gibco, Carlsbad, CA, USA) supplemented with 10% fetal bovine serum (Gibco). Let-7f-5p and let-7g-5p mimics (dsRNA oligonucleotides) and inhibitors (single-stranded chemically modified oligonucleotides) (GenePharma, Shanghai, China) were used for the in vitro overexpression and inhibition of let-7f-5p and let-7g-5p activity, respectively in S1 Table. Cells were plated in 6-well plates and cultured to 50–70% confluence prior to transfection. Lipofectamine LTX (Invitrogen, Carlsbad, CA, USA) was used for transfection according to the manufacturer’s instructions. Cells were transfected with RNAs at a final concentration of 10 nM; negative controls for the miRNA mimics and inhibitors were transfected as matched controls.

### Quantitative reverse transcription PCR

Total RNA (1 μg) from each sample was used for the reverse transcriptase (RT) reaction to generate cDNAs using a microRNA RT kit (Takara). Quantitative real-time PCR was performed (in triplicate) using a SYBR Green PCR kit (Takara) according to the manufacturer’s instructions with an ABI Prism 7300HT real-time PCR system (Thermo Fisher Scientific, MA, USA). The PCR primers used in the study are listed in S1 Table. The expression levels of miRNAs and the predicted target genes (*IGF2BP-1* and *IGF2R*) were normalized to those of the U6 snRNA and *ACTB*, respectively. Relative expression was calculated using the 2^-ΔΔCT^ method [22].

### Statistical analysis

All data are presented as the mean ± SD. The statistical significance of differences between experimental groups was assessed using the Student’s t-test or nonparametric test, and a P-value <0.05 was considered indicative of statistical significance.

## Results

### Profiles of miRNA expression in human early pregnancy decidua and menstrual endometrium

A total of 2,042 known miRNAs were expressed in the human decidua and endometrium samples analyzed. In addition, 177 miRNAs were differentially expressed between groups, including 88 that were up-regulated and 89 that were down-regulated (S2 Table). Clean sequence reads for the abundantly expressed miRNAs (>10,000 transcripts per million [TPM]) were used for further analyses. The known miRNAs in both groups exhibited a similar distribution in each library, based on classification by TPM. In normal decidua, 96.82% of miRNAs were expressed at low levels (<10 TPM), 3.04% were expressed at moderate levels (10–10,000 TPM), and only 0.15% of miRNAs were abundantly expressed (>10,000 TPM). However, the abundantly expressed miRNAs accounted for 87.11% of all miRNA reads. In the terminated-pregnancy decidua, 97.01% of miRNAs were expressed at <10 TPM, 2.84% were expressed at 10–10,000 TPM, and only 0.15% of miRNAs were expressed at >10,000 TPM. The abundantly expressed miRNAs accounted for 87.6% of all miRNA reads.

**Fig 1:**
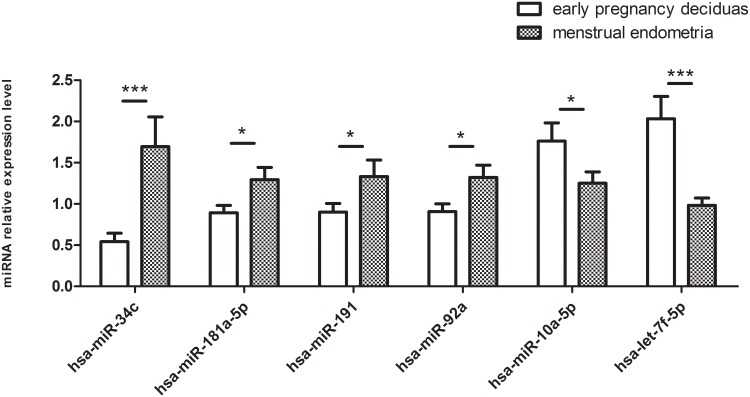
Validation of the differential expression of six known miRNAs by qPCR analysis. The miRNA expression level was normalized to that of U6 snRNA. “*” represents P value <0.05, “**” represents P value <0.01, and “***” represents P value <0.001. All experiments were repeated with 3 replicates.

The three most abundantly expressed miRNAs in both groups were hsa-let-7f-5p, hsa-miR-199a-3p, and hsa-miR-199b-3p, accounting for 73.28%, 6.91%, and 6.91% of the miRNome, respectively. The differential expression of six abundantly expressed miRNAs (miR-34c, miR-92a, miR-181a-5p, miR-191, miR-10a-5p, and let-7f-5p) was verified by qRT-PCR analysis of the 35 normal-pregnancy deciduas and 25 menstrual endometria (Fig 1). In addition, qPCR analysis confirmed the differential expression of four significantly down-regulated miRNAs and two significantly up-regulated miRNAs in the normal-pregnancy deciduas examined.

### Discovery of novel miRNAs

A total of 62 novel miRNAs were some identified in deciduas and some in the menstrual endometria (Table 1). The expression of four novel miRNAs (miR-1, miR-12, miR-18, and miR-110) was markedly altered in the decidua, but these miRNAs were expressed at low levels (data not shown).

**Table 1:** The 62 novel miRNAs expressed in the human decidua and menstrual endometrium. Candidates with sequence overlap with known miRNAs (but having a distinct mature miRNA sequence: isomiRs) and sequence alignments of novel miRNA candidates with known miRNAs of other species are shown.

			Reads (Normalized)		
Novel miRNA	Sequence	Chromosome	early pregnancy	menstrual endometrium	P_value
novel_mir_126	CCTGGAGAGGCTTGGTATGAGCG	chr6:168714989:168715066:-	0	1.3	0.36
novel_mir_131	CAGAGGCGTCGCTGGTCTTT	chr1:149711776:149711867:-	0	1.21	0.36
novel_mir_73	TTGAGGGGAGAATGAGGTGGAGA	chr1:43830309:43830391:-	2.7	12.18	0.11
novel_mir_117	GCCGGACAAGAGGGAGGTGAC	chr3:25706352:25706437:-	0	1.69	0.36
novel_mir_48	GAGAACCTGGGCAGAAGCTGAT	chr5:64332325:64332403:+	7.6	2.92	0.34
novel_mir_110	CTCCTGCGTAGGATCTGAGGAGT	chr20:30194978:30195067:+	0	6.14	0.03
novel_mir_11	GGAGGAACCTTGGAGCTTCGGCA	chr22:31556037:31556127:-	55.7	49.63	0.41
novel_mir_8	TCACGTCCCTGTTCGGGCGCCA	chr19:58024375:58024442:-	0	6.69	0.22
novel_mir_3	TGGGAGGAACAAGTATGCATT	chr11:16984501:16984581:-	54.4	20.58	0.16
novel_mir_125	AGATTGTAGTATGTGCATTGTT	chr2:208832756:208832839:-	0	3.38	0.36
novel_mir_4	AGGGGCGCGGCCCAGGAGCTCAG	chr11:125757935:125758025:-	0	1.19	0.36
novel_mir_124	CCGGGAGGGCAGTGGAGGCGTG	chr22:50747028:50747103:-	0	3.13	0.18
novel_mir_101	GCGGGCGGACGAGCGGGCGGGA	chr8:144897778:144897866:-	1.6	5.68	0.34
novel_mir_52	TCGGGGAGATGAGAGACGTGT	chr6:42071607:42071696:-	4.32	4.89	0.90
novel_mir_26	CCCTGGGGTTCTGAGGACAT	chr9:35710651:35710743:-	5.35	20.54	0.24
novel_mir_34	CCCTGGGGTTCTGAGGACATG	chr9:35710651:35710743:-	14.6	6	0.34
novel_mir_109	CTGTGGGCTCCTGGGATGGTTCT	chr1:17215841:17215936:-	0	3.08	0.17
novel_mir_87	AACACTGAAGTTAATGGCTGAT	chr2:85935673:85935771:-	11.2	12.59	0.92
novel_mir_129	AACTGGGCATAGCTGTACTTTT	chr8:141538823:141538913:-	0	2.62	0.36
novel_mir_114	AAGGGAAAGGGTCGATTGGGT	chr17:7754252:7754345:+	0	0.84	0.36
novel_mir_115	CCGGCCAACGCAGTGTCAGTTTT	chr1:145397811:145397885:-	0	1.18	0.36
novel_mir_116	TGGGCCTTGCTCTGGAGAGGCAG	chr1:150533203:150533277:-	0	0.84	0.36
novel_mir_119	GCAGGGAACACAGTACGGCTTG	chr12:125424204:125424296:-	0	2.61	0.36
novel_mir_113	TCAGACACAGGTATGGCTGGCTCC	chr5:171831829:171831908:-	0	3.29	0.36
novel_mir_128	CAGGATGTGGGTGGTGGCGGGTG	chr15:93541625:93541715:+	0	1.31	0.36
novel_mir_62	GCAAAATGATGAGGTACCTGATA	chr20:3194750:3194836:+	0	5.16	0.22
novel_mir_123	CCGGTCGCCGCGGTTCGCCGCC	chrUn_gl000220:156679:156752:+	0	3.79	0.36
novel_mir_123	CCGGTCGCCGCGGTTCGCCGCC	chrUn_gl000220:112707:112780:+	0	3.79	0.36
novel_mir_19	GAGGATGACGCAGACTTGGT	chr6:5148472:5148552:+	31.2	2.76	0.13
novel_mir_120	TGACACTGTGGGGAACGTGCA	chr16:30198328:30198410:+	0	1.42	0.36
novel_mir_51	TCGGGCGGGAGTGGTGGCTTT	chr6_mann_hap4:222225:222307:+	1393	265	0.09
novel_mir_51	TCGGGCGGGAGTGGTGGCTTT	chr6_qbl_hap6:222220:222302:+	1393	265	0.09
novel_mir_51	TCGGGCGGGAGTGGTGGCTTT	chr6:28918820:28918902:+	1393	265	0.09
novel_mir_51	TCGGGCGGGAGTGGTGGCTTT	chr6_ssto_hap7:259727:259809:+	1393	265	0.09
novel_mir_51	TCGGGCGGGAGTGGTGGCTTT	chr6_mcf_hap5:222373:222455:+	1393	265	0.09
novel_mir_51	TCGGGCGGGAGTGGTGGCTTT	chr6_cox_hap2:437556:437638:+	1393	265	0.09
novel_mir_21	TGAGCACCCCAGGACCTGCGCT	chr7:100240307:100240383:-	0	2.7	0.18
novel_mir_118	CACTGGCATTAGTGGGACTTTT	chr3:184970973:184971071:-	0	0.84	0.36
novel_mir_121	CTGGGAGGCGGTCGGTTCTGAG	chr19:1253351:1253436:+	0	1.19	0.36
novel_mir_122	CGCGAGAAGAGGCTGAACTG	chr1:28573676:28573749:-	0	1.9	0.36
novel_mir_38	CACAGGCAGGAGACCCCACA	chr3:53797063:53797141:+	0	1.9	0.36
novel_mir_108	TCAGGTAGTAGGGCATTGTGGTT	chr14:55587747:55587820:+	0	1.26	0.36
novel_mir_23	TGAGTGTGTGTGTGTGAGTGTGA	chr8:79679467:79679541:+	20.7	18.92	0.79
novel_mir_12	AACACTGAAGTTAATGGCTGA	chr2:85935674:85935771:-	0	48.52	0.04
novel_mir_83	CTGTGGGCTCCTGGGATGGTT	chr1:17215843:17215929:-	6.55	1.52	0.18
novel_mir_33	TCGGGGAGATGAGAGACGTGTT	chr6:42071607:42071696:-	3.22	1.3	0.47
novel_mir_130	AGGGGCGCGGCCCAGGAGCTCAGA	chr11:125757934:125758033:-	0	1.21	0.36
novel_mir_112	TCTCACCTGGCATAAGCAATT	chr5:167592851:167592932:+	0	1.26	0.36
novel_mir_127	TCTTGGAATGGCACAGCTGAGA	chr10:3436634:3436717:+	0	2.62	0.36
novel_mir_17	TCAGACACAGGTATGGCTGGCT	chr5:171831829:171831908:-	0	2.7	0.36
novel_mir_32	CCGGTCAGGGAATGAGGTTTT	chr6_qbl_hap6:253423:253493:+	4.9	8.45	0.64
novel_mir_32	CCGGTCAGGGAATGAGGTTTT	chr6_mann_hap4:253429:253499:+	4.9	8.45	0.64
novel_mir_32	CCGGTCAGGGAATGAGGTTTT	chr6_dbb_hap3:253437:253507:+	4.9	8.45	0.64
novel_mir_32	CCGGTCAGGGAATGAGGTTTT	chr6_cox_hap2:468829:468899:+	4.9	8.45	0.64
novel_mir_32	CCGGTCAGGGAATGAGGTTTT	chr6:28950026:28950096:+	4.9	8.45	0.64
novel_mir_32	CCGGTCAGGGAATGAGGTTTT	chr6_ssto_hap7:290997:291067:+	4.9	8.45	0.64
novel_mir_32	CCGGTCAGGGAATGAGGTTTT	chr6_apd_hap1:253428:253498:+	4.9	8.45	0.64
novel_mir_25	AGGGCCGAAGGGTGGAAGCTG	chr9:135927378:135927447:+	0	2.78	0.36
novel_mir_64	ATGGGGACAGGGATCAGCATG	chr2:219206629:219206708:+	0	3.32	0.36
novel_mir_132	CAGGGCTGGGAGGGAGTGGGA	chr1:153585537:153585627:-	0	1.45	0.36
novel_mir_14	GCTGGCTCGCGATGTCTGTTT	chr3:45730469:45730561:-	49.6	118.58	0.20
novel_mir_97	AAGGTAGCGGGAGGGTTGGGCT	chr3:45883723:45883820:+	1.6	3.52	0.42
novel_mir_20	TCGGGCGGGAGTGGTGGCTTTT	chr6_ssto_hap7:259726:259810:+	473	1109.78	0.30
novel_mir_20	TCGGGCGGGAGTGGTGGCTTTT	chr6_cox_hap2:437555:437639:+	473	1109.78	0.30
novel_mir_20	TCGGGCGGGAGTGGTGGCTTTT	chr6_mcf_hap5:222372:222456:+	473	1109.78	0.30
novel_mir_20	TCGGGCGGGAGTGGTGGCTTTT	chr6_qbl_hap6:222219:222303:+	473	1109.78	0.30
novel_mir_20	TCGGGCGGGAGTGGTGGCTTTT	chr6_mann_hap4:222224:222308:+	473	1109.78	0.30
novel_mir_20	TCGGGCGGGAGTGGTGGCTTTT	chr6:28918819:28918903:+	473	1109.78	0.30
novel_mir_100	ACAAGAGAGCAGAACGAGGTT	chr6:6152173:6152249:-	2.06	1.66	0.88
novel_mir_28	TTGTGGAAACAATGGTACGGCA	chr15:45493361:45493452:+	28.6	18.18	0.49
novel_mir_15	ATGGGGAGGTGTGGAGTCAGCAT	chr3:127294107:127294180:-	2.06	2.57	0.85
novel_mir_82	TCCTGGAGCTGGGCAGATGGGA	chr19:2762628:2762702:-	1.31	2.82	0.51
novel_mir_111	TGGGGAGGTGTGGAGTCAGCATG	chr3:127294100:127294179:-	0	3.03	0.36
novel_mir_133	AGGGTGTAGAAGAGAAGAAGAA	chr5:137810882:137810978:-	0	2.9	0.36
novel_mir_30	AGGGTCTGCAGGAAGGTCGGCT	chr2:113994290:113994372:-	40.2	8.9	0.35
novel_mir_24	AAACTGGGCATAGCTGTACTTTT	chr8:141538823:141538913:-	5.36	13.11	0.36
novel_mir_74	TGCCCGGCGGTGTGCGGCCACA	chr20:17486149:17486232:+	1.57	6.45	0.11
novel_mir_1	TGTGTTTGTGTATGTGTATGT	chr5:57745034–57745054:-	16.5464	0	0.01
novel_mir_18	GATGAGGAGGATGAGGAGGATG	chr3:51385333–51385354:+	10.0535	0	<0.01

### Target gene prediction and pathway analysis

**Fig 2:**
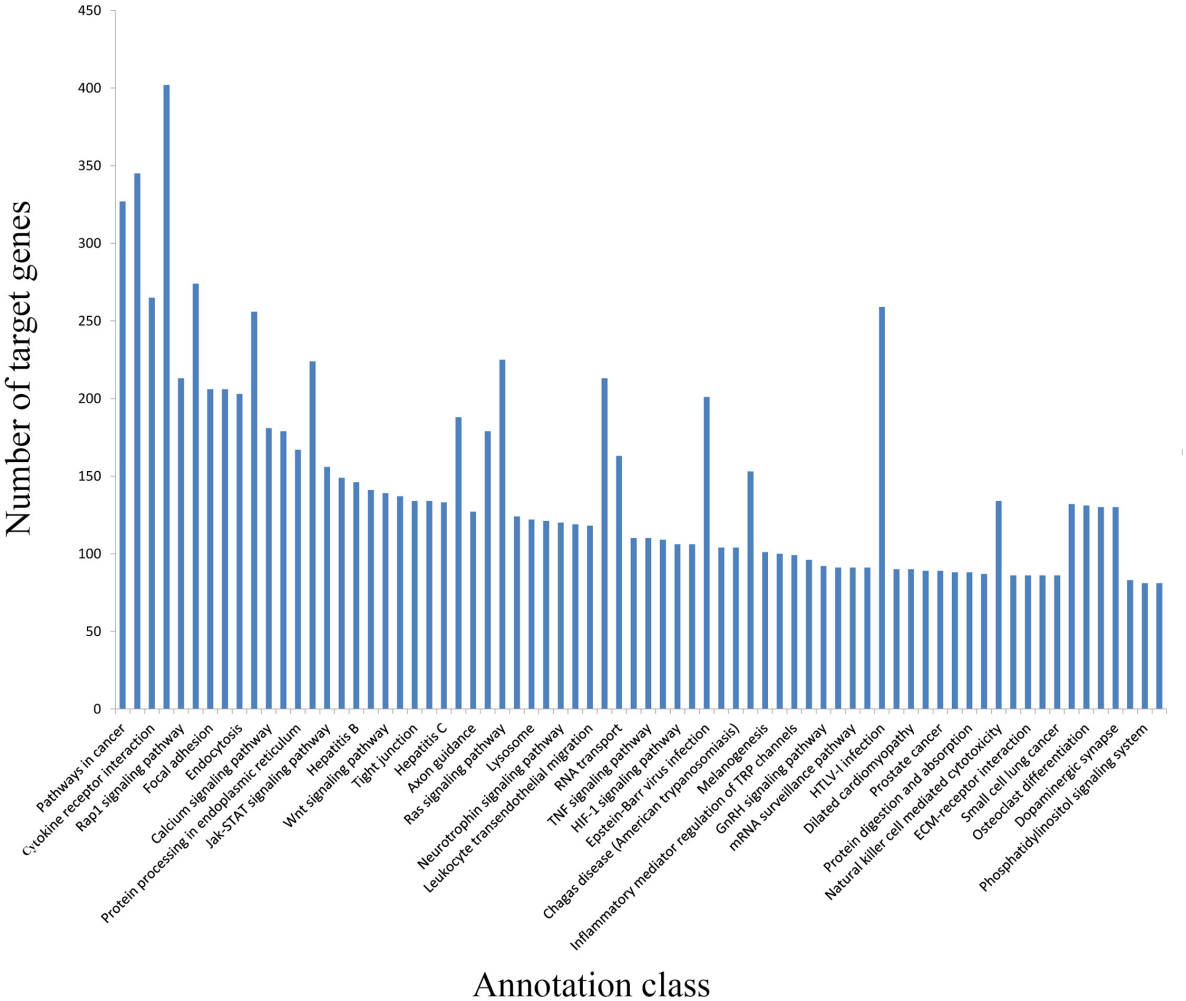
Kyoto Encyclopedia of Genes and Genomes analysis indicating the number of miRNA-targeted genes and the various associated pathways. Horizontal axis indicates the annotated signaling pathways identified, and the vertical axis indicates the number of genes in each pathway to which miRNAs were predicted to bind.

A total of 37,513 target genes were predicted using Mireap software. The majority of the target genes were found to be involved in cellular processes, regulation of biological processes, and metabolic processes. The pathways with the most genes targeted by the identified miRNAs as determined by KEGG analysis are shown in Fig 2.

### Target gene binding-site prediction

**Fig 3:**
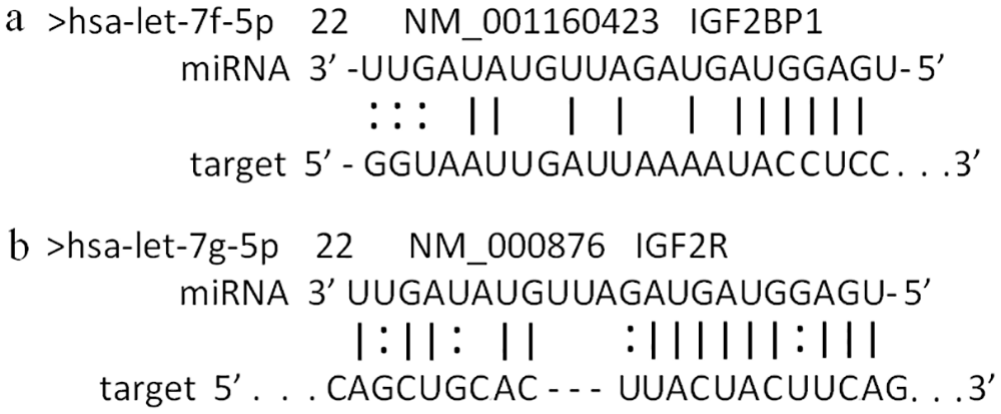
(a) Let-7f-5p was predicted to bind to a site on the *IGF2BP-1* mRNA. (b) Let-7g-5p was predicted to bind to a site on the *IGF-2R* mRNA.

A number of candidate miRNA-target genes involved in establishing and maintaining pregnancy were identified in previous studies [11, 23, 24]. These binding-site predictions from our analyses suggest that interactions between miRNAs and mRNAs are involved in the maintenance of early pregnancy (Fig 3).

### Changes in *IGF2BP-1* and *IGF2R* expression in vitro after overexpression or knockdown of let-7f-5p and let-7g-5p

**Fig 4:**
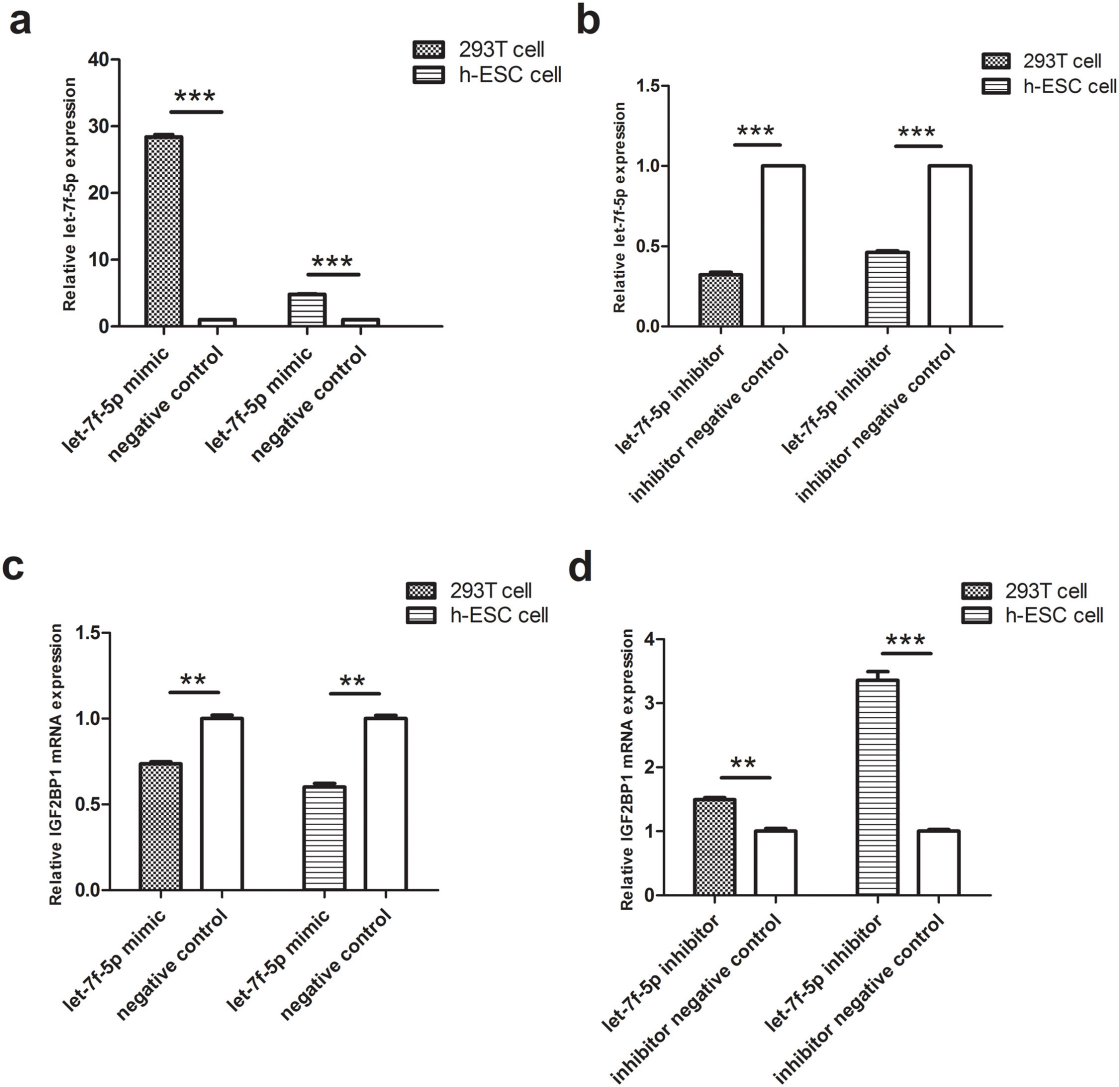
IGF2BP-1 expression in vitro after overexpression or knockdown of let-7f-5p. (a) Overexpression of let-7f-5p in HEK293T and hESCs cells. Cells were cultured and incubated with hsa-let-7f-5p mimic or negative control. Levels of let-7f-5p were determined using qPCR. (b) Knockdown of let-7f miRNA by transfection with hsa-let-7f-5p inhibitor. Cells were cultured and incubated with hsa-let-7f-5p inhibitor or negative control. Levels of let-7f-5p were determined using qPCR. (c-d) Expression of *IGF2BP-1* mRNA, as determined using qPCR analysis. Experiments were replicated three times, and error bars represent standard error. **P<0.01, ***P<0.001.

**Fig 5:**
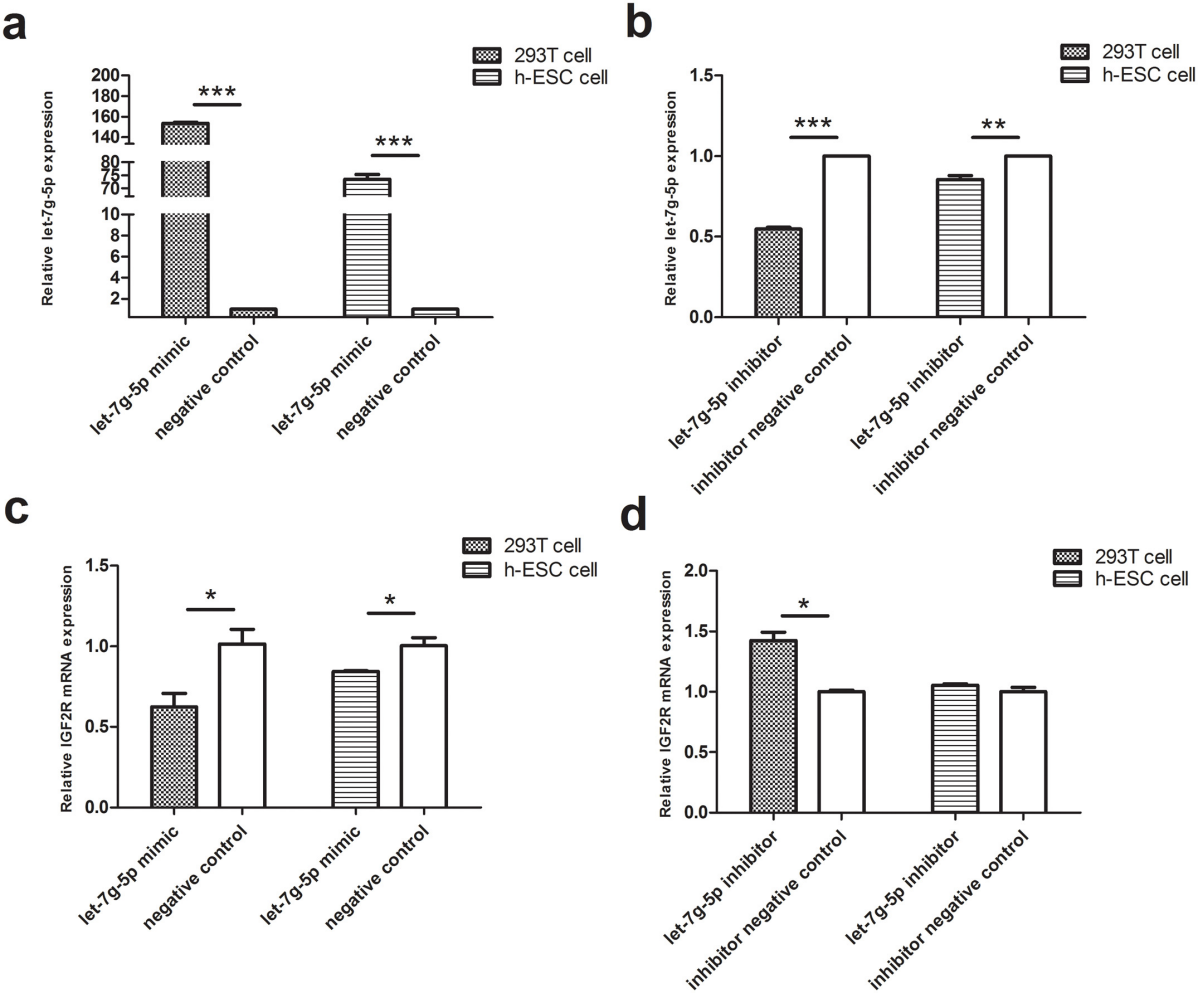
IGF2R expression in vitro after overexpression or knockdown of let-7g-5p. (a) Overexpression of let-7g-5p in HEK293T and hESCs cells. Cells were cultured and incubated with let-7g-5p mimic or negative control. Levels of let-7g-5p were determined using qPCR. (b) Knockdown of let-7g-5p miRNA by transfection with hsa-let-7g-5p inhibitor. Cells were cultured and incubated with hsa-let-7g-5p inhibitor or negative control. Levels of let-7g-5p were determined using qPCR. (c-d) Expression of *IGF2R* mRNA, as determined using qPCR analysis. Experiments were replicated three times, and error bars represent standard error. *P<0.05, **P<0.01 ***P<0.001.

To further investigate the effect of let-7 miRNA on decidualization, the expression of target genes was evaluated following overexpression or knockdown of the miRNAs mediated by corresponding miRNA mimics or inhibitors, respectively. Because miRNAs of the let-7 family were found to be abundantly expressed in decidua in a previous study [11], our in vitro experiments had focused solely on the effects of let-7f and let-7g-5p. In the present study, we found that the expression of *IGF2BP-1* mRNA increased in HEK293T and hESCs cells transfected with let-7f-5p inhibitor and decreased in cells transfected with let-7f-5p mimic (Fig 4). Likewise, the expression of *IGF2R* mRNA increased in cells transfected with let-7g-5p inhibitor and decreased in cells transfected with let-7g-5p mimic (Fig 5).

## Discussion

Decidualization involves the transformation of endometrial stromal fibroblasts into specialized secretory decidual cells that provide the nutritive and immuno-privileged matrix essential for embryo implantation and placental development. In contrast to the case with most mammals, embryo implantation is not required for decidualization of the human endometrium. A growing body of evidence suggests that specific miRNAs play roles in the development and progression of early pregnancy via the regulation of a wide variety of signaling pathways in the decidua, including those involved in mediating inflammation [25], local estrogen biosynthesis [26], progesterone resistance [8], endometrial stromal cell invasiveness [27], extracellular matrix remodeling [28], angiogenesis [29, 30], and epigenetic regulation [7].

In the present study, we investigated the miRNome of the normal-pregnancy decidua and menstrual endometrium (consisting of the decidualizing superficial endometrium, with decidualization triggered by a decline in the level of circulating progesterone). We identified 2,042 known miRNAs expressed in the samples of human decidua. A total of 177 miRNAs were differentially expressed between groups, 88 of which were up-regulated and 89 of which were down-regulated. In early pregnancy deciduas, 0.15% of the miRNAs were abundantly expressed (>10,000 TPM), and these miRNAs accounted for 87.11% of all miRNA reads. Similarly, in menstrual endometria, 0.15% of the miRNAs were abundantly expressed (>10,000 TPM), and these miRNAs accounted for 87.6% of all miRNA reads. The differential expression of six abundant miRNAs in deciduas from the terminated-pregnancy group was confirmed in further analyses.

In this study, we found that the expression of let-7f-5p was higher in the early pregnancy decidua than in the menstrual endometrium. However, little is currently known about the function and role of miRNAs of the let-7 family in the decidua, and no reports of the significance of let-7 involvement in embryo implantation have been published. Our previous study showed that let-7 family members were expressed abundantly in decidua and their down regulation in normal pregnancy might involve in decidualization comparing to the aborted decidua [11]. And some of let-7 family members also differentially expressed in decidua and menstrual endometrium. To investigate the function of let-7 family members, further study was performed. Hu et al [31] analyzed miRNA expression in mouse uterus using miRNA microarrays and showed that let-7a, let-7b, let-7c, let-7d, let-7e, let-7f-5p, let-7g-5p, and let-7i are highly expressed in IMs (inter-implantation sites). Lozoya [32] showed that let-7a miRNA expression was maximal in 6-week embryonic tissue, and abruptly dropped in the transition to week-7, to remain low in expression up to week-9.

These results suggest that let-7a may be involved in ectopic implantation. A study by Chan et al [33] showed that the expression of let-7b and let-7c is significantly lower in the choriodecidua compared with the placenta and amnion, whereas the expression of let-7d and let-7f is lower in the amnion relative to the choriodecidua and placenta. In addition, although miRNAs of the let-7 family are highly expressed in mature oocytes and zygotes, their expression declines in the two-cell stage [34]. Hence, the roles of let-7 miRNAs in decidualization at pregnancy initiation require further clarification.

Given their apparently crucial roles in establishing endometrium receptivity and in mediating events such as spontaneous miscarriage, we therefore sought to identify target genes by overexpression or knockdown of let-7 miRNA expression in vitro. We demonstrated for the first time that there is a significant inverse relationship between the expression of let-7 miRNAs and target genes. In mice, the Igf2r gene exhibits imprinted expression in post-implantation tissues coinciding with expression of Airn ncRNA[35], it has been demonstrated that bovine IGF2R also exhibits imprinted expression in post-implantation tissues[36]. The observed expression of bAIRN in only 11% of bovine conceptuses at Day 15 and 80% of conceptuses at Day 18 of gestation supports the suggestion that imprinted expression of IGF2R/AIRN is being established during the peri-implantation period, around the time of maternal recognition of pregnancy in cattle [37]. The expression of PDGFA, placenta growth factor, IGF2BP1 and IGF2BP3 were up-regulated in human trophectoderm cells in day 5 after human chorionic gonadotrophin administration, but their acceptors, such as PDGFRA and KDR were over-expressed in the receptive endometrium[38]. As *IGF2BP1* and *IGF2R* [39] are known to be involved in pregnancy establishment and maintenance [40], we hypothesized that let-7 disrupts decidualization from a maternal aspect by down-regulating the expression of *IGF2BP1* and *IGF2R*.

## Conclusion

In summary, this study characterized the miRNomes of the human decidua and menstrual endometrium during pregnancy, providing new data that should prove useful in subsequent investigations of the roles played by miRNAs during decidualization from a maternal aspect. Our in vitro data reveal an inverse relationship between the expression of the abundant let-7 miRNAs and their target genes, suggesting that miRNAs are involved in mediating endometrial receptivity and decidualization.

## Supporting Information

S1 Table Description of miRNA primers used for real-time RT-PCR. (DOCX)

S2 Table 177 Differentially expressed miRNAs in the human menstrual endometrium and early pregnancy decidua. (XLSX)
