# Supplementary material for: MicroRNA Profiles in Spontaneous Decidualized Menstrual Endometrium and Early Pregnancy Decidua with Successfully Implanted Embryos
Source: PLoS One. 2016 Jan 6;11(1):e0143116. doi: 10.1371/journal.pone.0143116 (PMC4703305; doi:10.1371/journal.pone.0143116)
Supplement: S1 Table — (DOCX) [file pone.0143116.s001.docx]

S1 Table a. Mimics, inhibitor, and controls sequences of hsa-miR-7f-5p and hsa-miR-7g-5p used in this study

| Oligos | Sequence | |
| --- | --- | --- |
| hsa-miR-7f-5p mimics | UGAGGUAGUAGAUUGUAUAGUUCUAUACAAUCUACUACCUCAUU | |
| hsa-miR-7f-5p inhibitor | AACUAUACAAUCUACUACCUCA |  |
| hsa-miR-7g-5p mimics | UGAGGUAGUAGUUUGUACAGUUCUGUACAAACUACUACCUCAUU | |
| hsa-miR-7g-5p inhibitor | AACUGUACAAACUACUACCUCA | |
| mimic Negative Control | UUCUCCGAACGUGUCACGUTTACGUGACACGUUCGGAGAATT | |
| inhibitor Negative Control | CAGUACUUUUGUGUAGUACAA | |

S1 Table b. Summary of miRNA primers used in real-time RT-PCR

| **Primer** | **Sequences (5’→3’)** | **Length (nt)** | **GC (%)** | **Tm** |
| --- | --- | --- | --- | --- |
| U6 | CAAGGATGACACGCAAATTCG | 21 | 47.6 | 69 |
| hsa-miR-34c | CGAGGCAGTGTAGTTAGCTGATTGCA | 26 | 50.0 | 65.1 |
| hsa-miR-181a-5p | CAACATTCAACGCTGTCGGTGAGTAAAA | 28 | 42.9 | 64.9 |
| hsa-miR-191 | CCAACGGAATCCCAAAAGCAGCTG | 24 | 54.2 | 65.1 |
| hsa-miR-92a | CTATTGCACTTGTCCCGGCCTGTAA | 25 | 52.0 | 64.9 |
| hsa-miR-10a-5p | CCGGTACCCTGTAGATCCGAATTTGTG | 27 | 51.9 | 65.2 |
| hsa-let-7f-5p | GCGCGCTGAGGTAGTAGATTGTATAGTTAAA | 31 | 41.9 | 65 |
